# Pericoronary radiomics texture features associated with hypercholesterolemia on a photon-counting-CT

**DOI:** 10.3389/fcvm.2023.1223035

**Published:** 2023-10-30

**Authors:** Jannik Kahmann, Hishan Tharmaseelan, Philipp Riffel, Daniel Overhoff, Theano Papavassiliu, Stefan O. Schoenberg, Matthias F. Froelich, Isabelle Ayx

**Affiliations:** ^1^ Department of Radiology and Nuclear Medicine, University Medical Center Mannheim, Heidelberg University, Mannheim, Germany; ^2^ Department of Diagnostic and Interventional Radiology and Neuroradiology, Bundeswehr Central Hospital Koblenz, Koblenz, Germany; ^3^ First Department of Medicine-Cardiology, University Medical Centre Mannheim, Heidelberg University, Mannheim, Germany

**Keywords:** photon-counting computed tomography, pericoronary adipose tissue, texture analysis, hypercholesterolemia, radiomics

## Abstract

**Introduction:**

Pericoronary adipose tissue (PCAT) stands in complex bidirectional interaction with the surrounding arteries and is known to be connected to many cardiovascular diseases involving vascular inflammation. PCAT texture may be influenced by other cardiovascular risk factors such as hypercholesterolemia. The recently established photon-counting CT could improve texture analysis and help detect those changes by offering higher spatial resolution and signal-to-noise ratio.

**Methods:**

In this retrospective, single-center, IRB-approved study, PCAT of the left and right coronary artery was manually segmented and radiomic features were extracted using pyradiomics. The study population consisted of a test collective and a validation collective. The collectives were each divided into two groups defined by the presence or absence of hypercholesterolemia, taken from self-reported conditions and confirmed by medical records. Mean and standard deviation were calculated with Pearson correlation coefficient for correlation of features and visualized as boxplots and heatmaps using R statistics. Random forest feature selection was performed to identify differentiating features between the two groups. 66 patients were enrolled in this study (34 female, mean age 58 years).

**Results:**

Two radiomics features allowing differentiation between PCAT texture of the groups were identified (*p*-values between 0.013 and 0.24) and validated. Patients with hypercholesterolemia presented with a greater concentration of high-density values as indicated through analysis of specific texture features as “gldm_HighGrayLevelEmphasis” (23.95 vs. 22.99) and “glrlm_HighGrayLevelRunEmphasis” (24.21 vs. 23.31).

**Discussion:**

Texture analysis of PCAT allowed differentiation between patients with and without hypercholesterolemia offering a potential imaging biomarker for this specific cardiovascular risk factor.

## 1. Introduction

Large technological improvements in the field of computed tomography (CT) have led to the recommendation of cardiac CT angiography (CCTA) as the initial diagnostic tool for patients with low to intermediate pretest probability for coronary artery disease (CAD) (1). Recently, it has even been outlined, that patients with stable chest pain and intermediate pretest probability of coronary artery disease and with similar risk for major adverse cardiovascular events (MACE) suffered from major procedure-related complications less frequently when receiving an initial CT compared to initial invasive coronary angiography (2).

In recent years it has become clear that CT images are more than just pictures and convey plenty of data not visible to the human eye (3). Radiomics as a method to extract and analyze this data is the next evolutionary step in medical image diagnostics. However, there are some limitations to radiomics analysis still keeping it from being part of clinical routines. In particular, it is still susceptible to changes in technical factors as for example the reconstruction algorithm, contrast, and layer thickness (4, 5).

The recently established photon-counting CT (PCCT) operates with smaller photon-counting detector (PCD) elements than conventional CT devices operating with energy-integrating detectors (EID) (6). These PCD elements directly convert every photon that hits the detector plate into an electrical impulse. PCCT thereby offers a better spatial resolution, higher signal-to-noise ratio, and lower beam hardening artifacts (6, 7). With these advantages, it provides high feature stability and may improve radiomics analysis and help overcome several of the limitations described above (8).

Pericoronary adipose tissue (PCAT) not only serves as a mechanical support for coronary arteries but has been shown to stand in complex bidirectional interaction with the embedded artery and is - when dysregulated - connected to vascular inflammation (9). In that case, the adipose tissue releases various pro-inflammatory and anti-vasodilation factors regulating vascular tension, cell proliferation, and cell migration, like adipokines, cytokines, and chemokines that unfold their inflammatory effect on the underlying vessel in an endocrine and paracrine manner (10, 11). It has even been shown that the expression of genes coding for some of these factors is upregulated in dysregulated PCAT of patients with CAD (12). Inflammation plays an essential role in many cardiovascular diseases including atherosclerosis, hypertension, and vascular aging (13). Therefore, structural changes of PCAT following a dysregulated homeostasis of coronary arteries and the surrounding PCAT can be seen as a risk factor for cardiovascular diseases and the development of major adverse cardiovascular events (14).

In this context, the pericoronary Fat Attenuation Index (FAI), defined as the mean CT attenuation of adipose tissue, has been shown to be a reliable marker for local inflammation (15). Furthermore, it was identified to be closely connected to the vulnerability of plaques in patients with non-ST-elevation acute coronary syndrome (16).

The above-described FAI already showed how analysis of PCAT in imaging can provide additional help in cardiac risk prediction and stratification. However, in this context more complex radiomics spatial and texture features are mostly not considered which could impact the FAI's ability to differentiate between various pathologies and cause an overlap of patients (17). Therefore, it can be of great value to study these complex features in patients with specific diseases with the goal of finding characteristic features as potential imaging biomarkers.

Cardiovascular disease (CVD), especially atherosclerotic cardiovascular disease, is still the leading cause of mortality globally (18). When it comes to the prevention of CVD and its consequences, identifying and treating modifiable risk factors such as hypercholesterolemia is of great importance (1). Although since the middle of the twentieth century, global declines in mortality from ischemic heart disease and stroke due to changes in health behavior and treatment of risk factors have been observed, there is still a lot of effort needed to tackle this significant burden for individual and public health (19). For this purpose, non-invasive detection of structural changes in PCAT of hypercholesterolemia patients may be helpful.

This study aimed to investigate if changes in PCAT texture not visible to the human eye of patients with hypercholesterolemia can be identified by texture analysis with PCCT, offering a potential imaging biomarker for the future.

## 2. Material and methods

### 2.1. Study design

Patients who presented with a clinical indication for contrast-enhanced cardiac CT according to the guidelines of the European Society of Cardiology (ESC) (1) were enrolled between April and July 2022 in this retrospective single-center study. Patients were excluded in case of a previous pacemaker or cardiac stent implantation. Additionally, patients were excluded in case of severe image artifacts, for example through motion artifacts. Clinical parameters were retrospectively extracted from an already available questionnaire regarding classical clinical risk factors. All investigations were conducted according to the Declaration of Helsinki. The study had institutional review board and local ethics committee approval (ID 2021-659).

### 2.2. Test patient collective

Based on inclusion and exclusion criteria, a total of 48 out of initially 56 CT scans of patients (20 males, 28 females, mean age 58 years, range: 38–82 years) were selected. All patients answered a detailed questionnaire regarding their cardiovascular risk factors. The *p*-values for age, sex, Agatston-score, and the risk factors hypertension, diabetes, and nicotine abuse were calculated. The patient population was then divided into two groups according to the presence of hypercholesterolemia taken from self-reported conditions and confirmed by medical records (20). 15 (7 male, 8 female) patients presented with hypercholesterolemia, and the other 33 (13 male, 20 female) patients without hypercholesterolemia defined the control group. According to the medical record, 9 (60.0%) patients already received specific medication to lower hypercholesterolemia. Three patients (20.0%) presented with elevated or high LDL cholesterol above 100 mg/dl (LDL cholesterol 120−391 mg/dl) under ongoing lipid lowering therapy. In 6 patients (40.0%) no additional information except for previously diagnosed hypercholesterolemia was found in the medical record. The patient characteristics are summarized in Table 1.

**Table 1:** Test patient collective overview. Mean and (SD) given for continuous variables.

Patient characteristics	Overall	Non-Hypercholesterolemia	Hypercholesterolemia	*p*-value
*n*	48	33	15	
Age	58.48 (9.99)	58.91 (11.42)	57.53 (5.95)	0.59
Sex m/f	20/28	13/20	7/8	0.65
Agatston Score	241.08 (615.11)	195.03 (495.91)	342.39 (831.91)	0.53
Stenosis > 50%	10	9	1	
Stenosis < 50%	74	54	20	
Hypertension (%)	25 (52%)	15 (45%)	10 (67%)	0.18
Diabetes (%)	8 (17%)	4 (12%)	4 (27%)	0.28
Nicotine (%)	26 (54%)	18 (55%)	8 (53%)	0.94

### 2.3. Validation patient collective

CT scans of 18 additional patients (12 males, 6 females, mean age 67 years, range: 29–87 years) were selected as a validation collective based on the same inclusion and exclusion criteria as the test patient collective. Nine of these patients presented with and nine without hypercholesterolemia and they were split into two groups accordingly. Again, the *p*-values for age, sex, Agatston-score and the risk factors hypertension, diabetes and nicotine abuse were calculated. The patient characteristics of the validation collective are summarized in Table 2.

**Table 2:** Validation patient collective overview. Mean and (SD) given for continuous variables.

Patients characteristics	Overall	Non-Hypercholesterolemia	Hypercholesterolemia	*p*-value
*n*	18	9	9	
Age	66.61 (11.58)	68.67 (10.80)	64.56 (11.96)	0.48
Sex m/f	12/6	6/3	6/3	1.00
Agatston Score	199.72 (324.79)	171.67 (330.93)	227.78 (327.75)	0.73
Stenosis > 50%	5	1	4	
Stenosis < 50%	39	23	16	
Hypertension (%)	14 (78%)	8 (89%)	6 (67%)	0.29
Diabetes (%)	5 (28%)	2 (22%)	3 (33%)	0.62
Nicotine (%)	6 (33%)	3 (33%)	3 (33%)	1.00

### 2.4. Cardiac CT imaging

All 66 patients were examined using a first-generation whole-body dual-source PCCT system (NAEOTOM Alpha; Siemens Healthcare GmbH, Forchheim, Germany). A prospective ECG-gated sequential mode was used, operating with a tube voltage of 120 kV for test-patients and automatic dose modulation with a CARE keV BQ setting of 64 as well as a gantry rotation time of 0.25 s. Due to software updates regarding the PCCT Scanner, the technical details of the scans of the validation patients differed slightly from those of the test patients. For validation scans a tube voltage of 140 kV was used.

For the reduction of heart rates to less than 65 beats per minute patients received 5–10 mg of *β*-blockers intravenously in absence of contraindications and in dependence on heart rate. Additionally, 0.8 ml of sublingual nitroglycerin was applied. A non-enhanced cardiac CT scan was performed on all patients to estimate coronary artery calcification. This was followed by the contrast-enhanced scan using 80 ml of iodine contrast (Iomeron 400, Bracco Imaging Deutschland GmbH, Konstanz, Germany) and a 20 ml saline chaser (NaCl 0.9%) with a weight-based flow rate of 5–6 ml/sec. The start of CCTA was determined by bolus tracking in the descending thoracic aorta.

### 2.5. Cardiac CT imaging analysis

**Figure 1:**
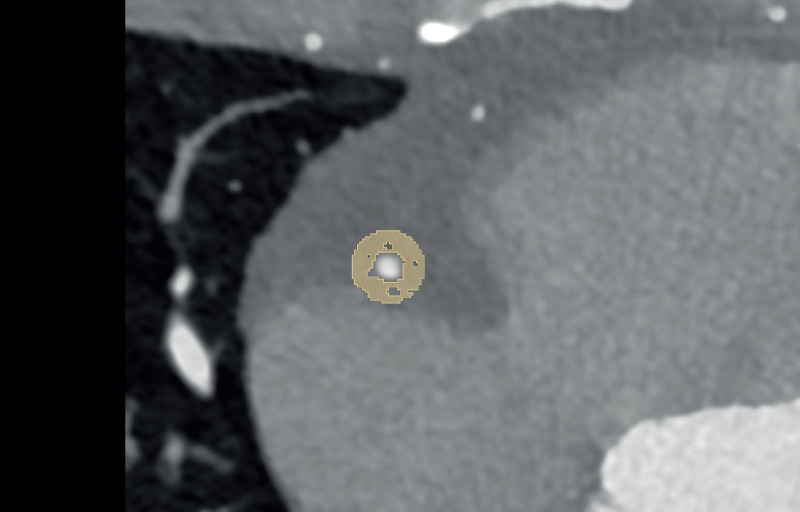
Example segmentation of pericoronary adipose tissue (in yellow) in a 39-year-old male on axial reconstructed images.

Estimation of coronary artery calcification was performed on axial non-enhanced scans with 3 mm slice thickness and Qr36 kernel using dedicated software (syngo.via, Siemens Healthcare GmbH, Forchheim, Germany). Axial images of contrast-enhanced CCTA were reconstructed using a slice thickness of 0.6 mm, an increment of 0.4 mm, and a soft vascular kernel (Bv40). These images were anonymized, exported, and stored in Digital Imaging and Communications in Medicine (DICOM) file format. Afterward, they were converted into Neuroimaging Informatics Technology Initiative (NIFTI) file format and uploaded into a dedicated segmentation tool (3D Slicer, Version 4.11) (21). The pericoronary adipose tissue was segmented manually by a medical student with one year of experience in segmentation and validated by a senior radiologist with ten years of experience in cardiothoracic imaging and six years of experience in segmentation. Pericoronary adipose tissue was defined as any voxel between −30 and −190 Hounsfield units (HU) surrounding the right coronary artery (RCA) or left anterior descending artery (LAD) along 6 cm starting 1 cm after the ostium of the RCA and along 4 cm directly after the bifurcation of the left main artery (LM). The segmentation was performed within a radial distance from the outer vessel wall equal to the diameter of the underlying vessel. Depending on the vessel, this radial distance was between 2 mm and 6 mm. Figure 1 shows an example segmentation of the pericoronary adipose tissue in an axial view.

### 2.6. Radiomics feature extraction and statistical analysis

Radiomics features shape, first-order, Gray Level Co-occurrence Matrix (glcm), Gray Level Dependence Matrix (gldm), Gray Level Size Zone Matrix (glszm), Gray Level Run Length Matrix (glrlm), and Neighboring Gray Tone Difference Matrix (ngtdm) were extracted from CT-scans of the test patient collective in pyradiomics (version 3.0.1) (22). The extracted features were imported into R Statistics (Version 4.2.0, R Core Team, Vienna, Austria) (23) and analyzed in RStudio (Version 2022.07.1 + 554, Boston, MA) (24) for statistical analysis. Mean and standard deviation values were calculated with Pearson correlation coefficient for correlation of features and all radiomics features were normalized using the z-score:

z=((X−μ))/σ

A clustered heatmap was generated using the ComplexHeatmap package for R illustrating the distribution of extracted features within both groups (Complementary Figure 1). As the ROI was identically determined for every segmentation as described above and the focus was on analyzing texture features, shape-based features were excluded before performing a permutation-based random forest (RF) classification with the Boruta package for R. Selected Features were visualized as boxplots. For validation purposes, radiomics features of the validation collective were extracted in the same way. The distribution of the previously selected features was then compared to their distribution within the validation collective.

## 3. Results

### 3.1. Cluster analysis

**Figure 2:**
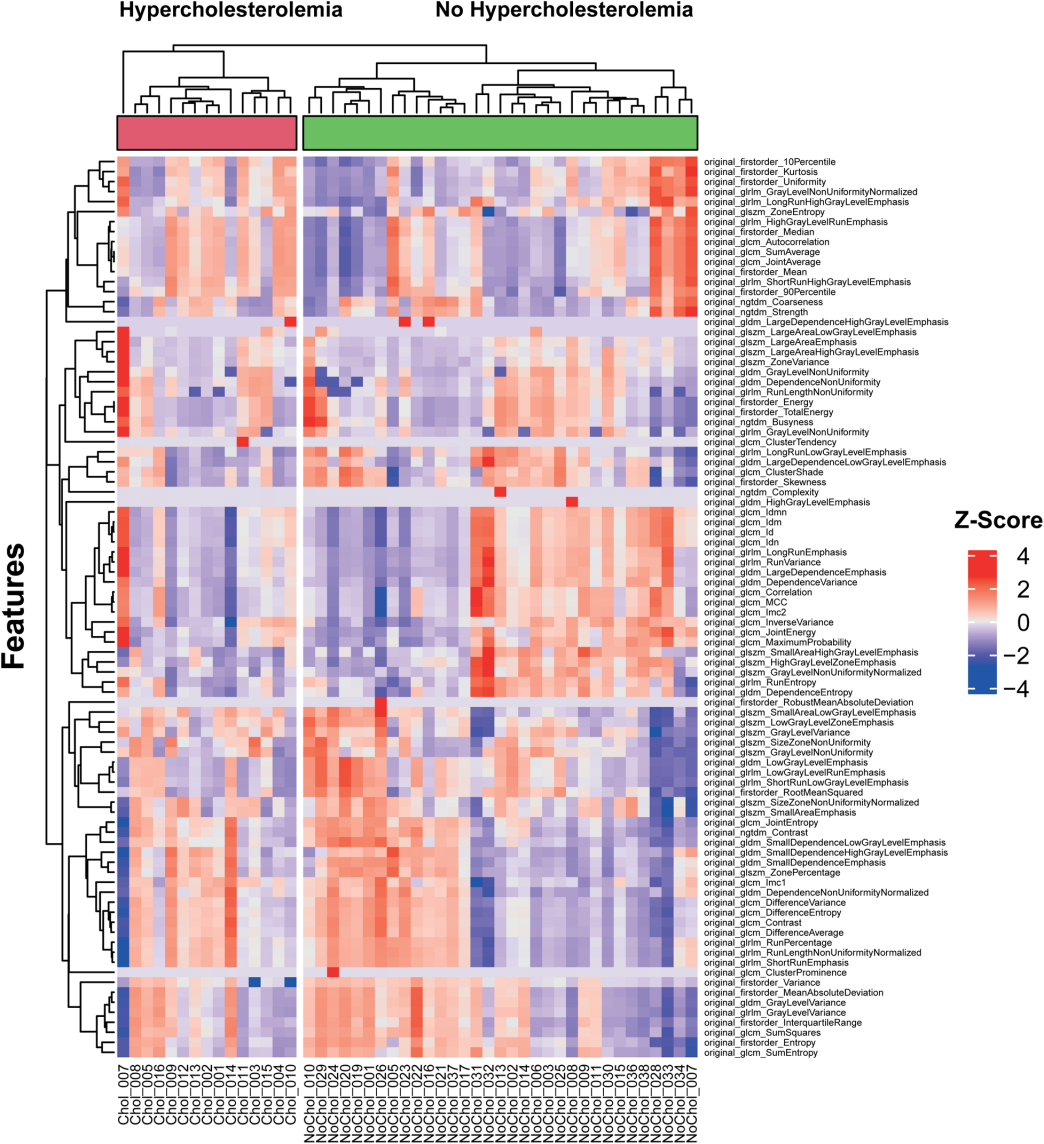
Unsupervised clustering heatmap of pericoronary adipose tissue radiomics features of the test collective.

K-means clustering of extracted radiomics features from the PCAT of each test patient was performed. Further clustering within the two groups of patients with and without hypercholesterolemia was added. The results are illustrated as a heatmap shown in Figure 2. The analysis showed a separation of features into three main clusters. Generally, the non-hypercholesterolemia group showed a higher variance with more features presenting with extremer values, while the hypercholesterolemia group overall displayed a more homogenous picture of rather intermediate values. Only one patient of the hypercholesterolemia group fell out of this pattern showing extreme values for almost every feature. Features in the first cluster showed mostly slightly positive z-score values combined with fewer slightly negative values for hypercholesterolemia patients while showing notably more negative or higher positive values for non-hypercholesterolemia patients. This cluster also stood out by consisting of many of the first-order features including “original_firstorder_Mean” and “original_firstorder_Median”. In the second cluster, more positive values were found within the non-hypercholesterolemia group compared to the hypercholesterolemia collective. Similar to the first cluster, the features of hypercholesterolemia patients in the third cluster had mostly values in the intermediate positive range. The non-hypercholesterolemia group presented with more negative and extreme values. Furthermore, features describing similar characteristics were mostly clustered together, as can be seen at the bottom of the figure, where different features representing variance and entropy including for example “original_firstorder_Variance”, “original_gldm_GrayLevelVariance” and “original_glcm_SumEntropy” showed extremely similar values within each patient. Non-hypercholesterolemia patients were separated into two main clusters. The biggest difference between these clusters could be found in the second and third clusters. In the second cluster, 14 out of 33 patients showed mostly negative values, while the rest presented with more positive features. In cluster three, this pattern was reversed, with the same 14 individuals having almost exclusively positive values, while values of the other 19 patients' features were predominantly negative. Within the hypercholesterolemia group, the differences between patients were less impressive with the exception of one patient.

### 3.2. Pericoronary adipose tissue radiomic signature of hypercholesterolemia

**Figure 3:**
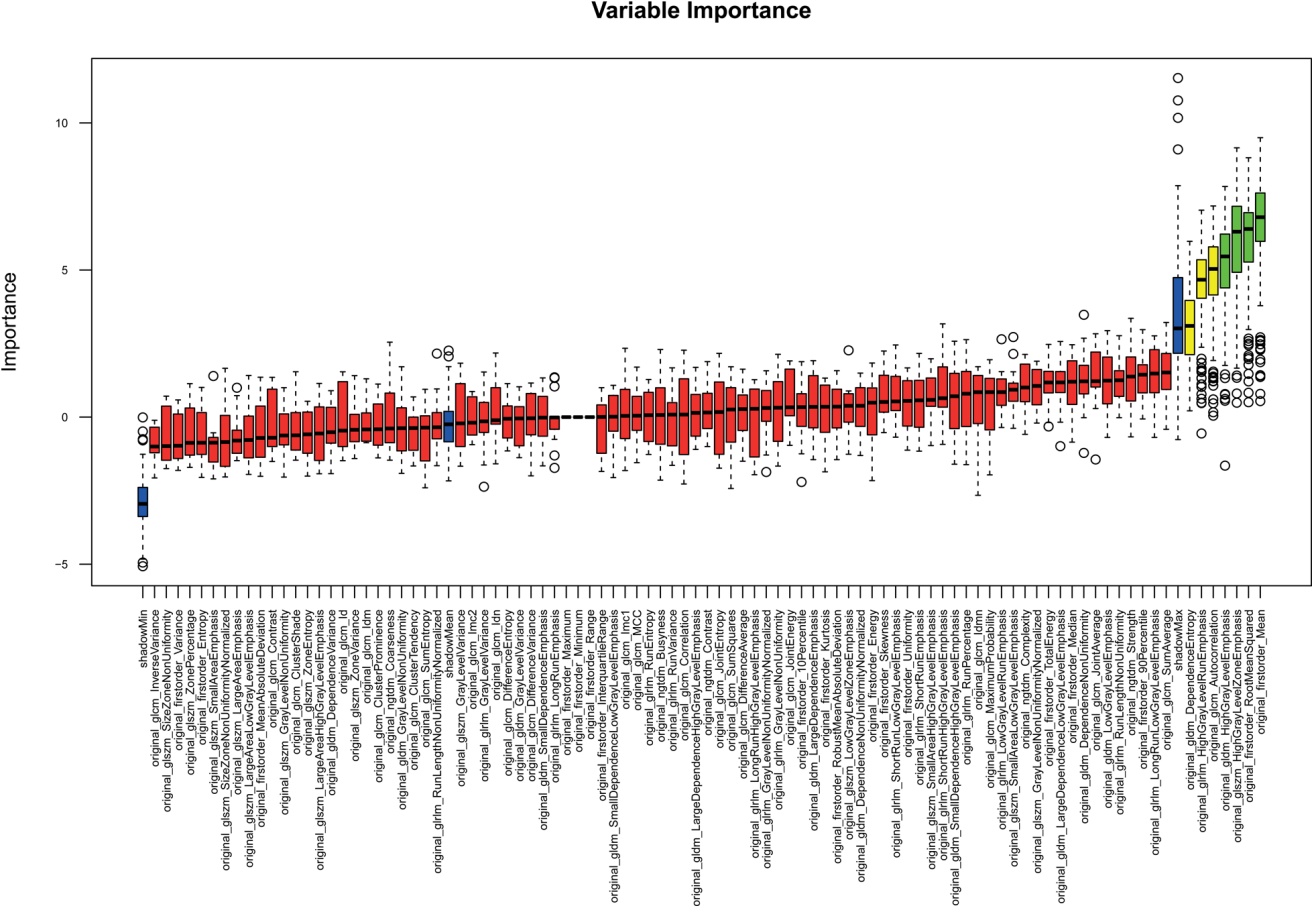
Random forest feature selection: features relevant for the differentiation of distinct subgroups are in green.

**Figure 4:**
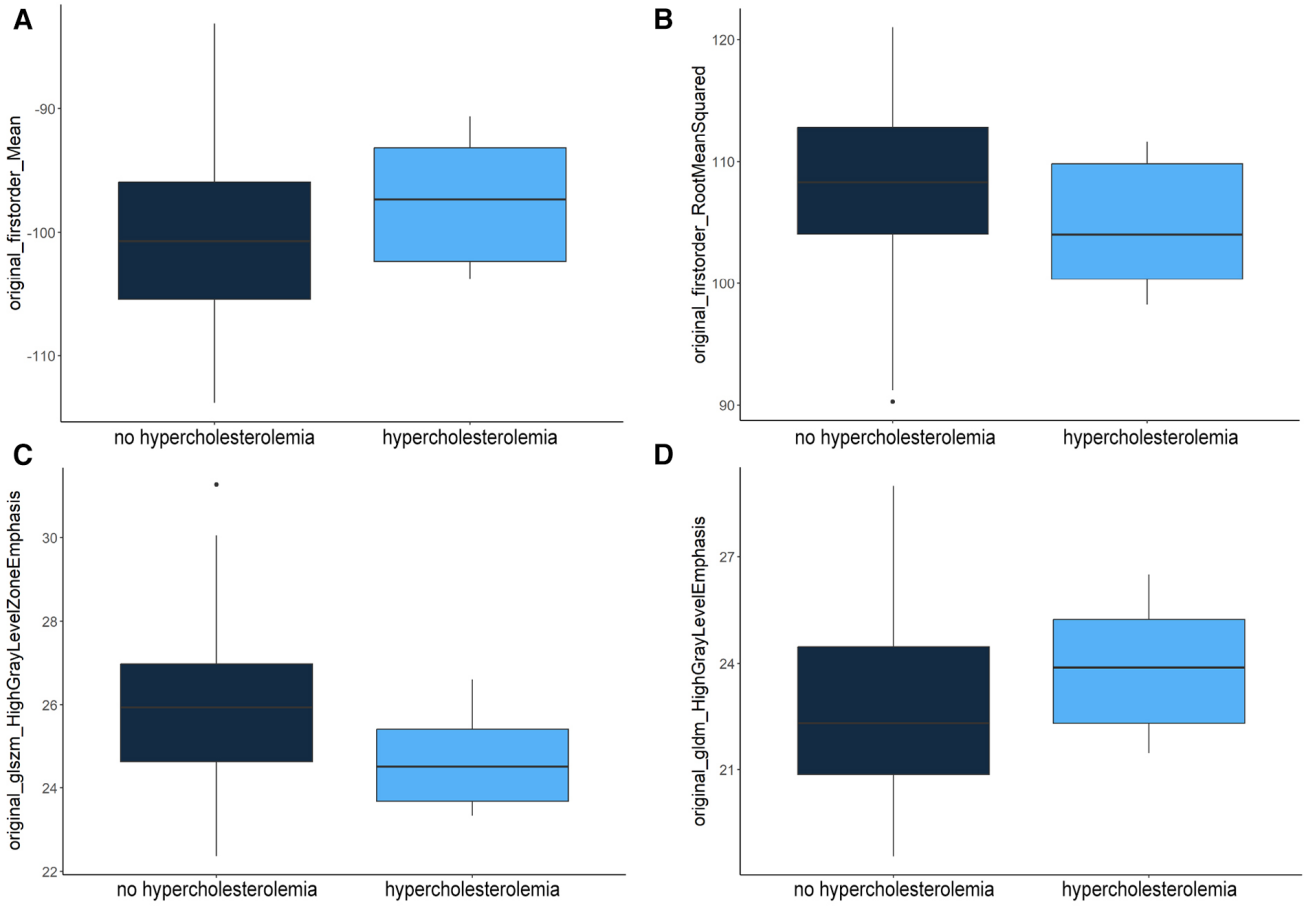
Distribution of “original_firstorder_Mean” (**A**), “original_firstorder_RootMeanSquared” (**B**), “original_glszm_HighGrayLevelZoneEmphasis” (**C**) and “original_gldm_HighGrayLevelEmphasis” (**D**) features within the test dataset.

RF feature selection was used to determine important features allowing a differentiation of patients based on PCAT texture. The algorithm was performed on patients with and without hypercholesterolemia. The results are visualized in Figure 3. This led to the selection of the following six features: “original_firstorder_Mean”, “original_firstorder_RootMeanSquared”, “original_gldm_HighGrayLevelEmphasis”, “original_glszm_HighGrayLevelZoneEmphasis”, “original_glcm_Autocorrelation”, and “orignal_glrlm_HighGrayLevelRunEmphasis” with the first four features of this list being of higher importance than the last two according to RF selection criteria (Table 3). These especially important features are visualized as boxplots (Figure 4).

**Table 3:** Selected radiomics features within the test collective. Mean and (SD) given for continuous variables.

	Non-Hypercholesterolemia	Hypercholesterolemia	*p*-value
Original_firstorder_Mean	−99.96 (7.92)	−97.09 (4.98)	0.20
Original_firstorder_RootMeanSquared	107.23 (7.72)	104.45 (5.02)	0.20
Original_glcm_Autocorrelation	20.58 (2.43)	21.43 (1.59)	0.22
Original_gldm_HighGrayLevelEmphasis	22.99 (2.81)	23.95 (1.73)	0.23
Original_glrlm_HighGrayLevelRunEmphasis	23.31 (2.73)	24.21 (1.66)	0.24
Original_glszm_HighGrayLevelZoneEmphasis	25.97 (1.86)	24.64 (1.14)	0.013

“Original_firstorder_Mean” representing the average gray level intensity within the region of interest (25) had a higher mean value of −97.09 in patients with hypercholesterolemia compared to patients without (−99.96). A similar distribution was observed for the two features measuring the concentration of high gray-level values in the image (25): “original_gldm_HighGrayLevelEmphasis” (23.95–22.99) and “original_glrlm_HighGrayLevelRunEmphasis” (24.21–23.31). On the other hand, “original_firstorder_RootMeanSquared” (104.45–107.23) and “original_glszm_HighGrayLevelZoneEmphasis” (24.64–5.97) had lower mean values in patients with hypercholesterolemia. “Original_firstorder_RootMeanSquared” provides information about the overall intensity of pixel values (their distance from zero in negative or positive direction) within the region of interest and “Original_glszm_HighGrayLevelZoneEmphasis” assesses the distribution of higher gray-level values, where a higher score signifies a larger presence of elevated gray-level values and size zones within the image (25). The *p*-values were calculated using the two-sample t-test assuming unequal variances. It should be noted that the feature “original_glszm_HighGrayLevelZoneEmphasis” showed the lowest *p*-value (0.013) despite not being selected as the most important feature through RF feature selection.

For validation, the distribution of the four most important selected features within the test group was compared to their distribution within the validation group. This distribution is shown in Table 4. The comparison showed, that while mean values of the two first-order-features “original_firstorder_Mean” (−101.06 in validation patients with hypercholesterolemia to −100.89 in patients without) and “original_firstorder_RootMeanSquared” (108.04–107.50) did not show the same tendencies in the validation group, the higher-order-features “original_glszm_HighGrayLevelZoneEmphasis” (25.79–26.22) and “original_gldm_HighGrayLevelEmphasis” (22.53–22.45) in fact showed the same tendencies. Both in the test collective and in the validation collective, “original_glszm_HighGrayLevelZoneEmphasis” had a lower mean value in scans of patients who presented with hypercholesterolemia while “original_gldm_HighGrayLevelEmphasis” was higher in those scans.

**Table 4:** Selected radiomics features within the validation collective. Mean and (SD) given for continuous variables.

	Non- Hypercholesterolemia	Hypercholesterolemia	*p*-value
Original_firstorder_Mean	−100.89 (8.53)	−101.06 (8.15)	0.97
Original_firstorder_RootMeanSquared	107.50 (8.44)	108.04 (7.77)	0.88
Original_glszm_HighGrayLevelZoneEmphasis	26.22 (0.93)	25.79 (1.50)	0.47
Original_gldm_HighGrayLevelEmphasis	22.45 (2.89)	22.53 (2.92)	0.96

## 4. Discussion

In this study, we demonstrated that radiomics-based texture features of PCAT of the left and right coronary arteries possibly differ between patients with and without hypercholesterolemia. Differentiation between the two patient groups was possible through the analysis of six different radiomics features. The differentiating power of the two higher-order features “original_glszm_HighGrayLevelZoneEmphasis” and “original_gldm_HighGrayLevelEmphasis” could be further validated by comparison of the test and the validation collective. PCAT of patients with hypercholesterolemia overall showed a higher mean density compared to the control group. In line with these results, they also showed a predominantly higher concentration of high-density areas possibly reflecting the inflammatory reaction in the pericoronary adipose tissue in patients presenting with hypercholesterolemia. This correlates well with a greater concentration of high-density areas in gray-level runs, which are defined as the length of a sequence of consecutive pixels with the same gray-level value (25). Through the inflammatory reaction, the PCAT adjacent to the vessel wall could be affected the most, which is reflected in the linear presentation of high gray levels. On the contrary, patients with hypercholesterolemia presented with a lower proportion of high-intensity values and size zones in the image. This disagreement could possibly reflect the coexisting of other cardiovascular risk factors with a potential underlying more diffuse inflammatory effect on the PCAT. Analysis of the k-means clustering of the features showed that the non-hypercholesterolemia group presented with a greater variety and more extreme values than the patients with hypercholesterolemia. The more fluctuating tissue texture within the non-hypercholesterolemia group could be due to different independent risk factors, while the more uniform texture within the hypercholesterolemia group could be explained by the common predominant effect of hypercholesterolemia on the PCAT.

Antonopoulos et al. described how the measurement of adipose tissue composition from CT images can be used as a noninvasive biomarker for vascular inflammation. Using the standardized adipose tissue CT attenuation and post-processing adjustments they developed the Fat Attenuation Index reflecting adipocyte differentiation and lipid accumulation. In particular, this index proved to be strongly associated with the presence of cardiovascular disease and to be able to separate stable from unstable lesions (15, 16). The results of our study are in line with these results indicating a higher mean density to correlate with hypercholesterolemia. In comparison to the results presented by Antonopoulos et al., our studies did not focus on pure tissue CT attenuation values but on a deeper texture analysis of the adipose tissue itself, offering potential additional imaging biomarkers in the future, as there is still discussion about the epicardial adipose tissue (EAT) density, as some authors describe a lower EAT density to correlate with cardiovascular risk and disease (26, 27).

Lin et al. showed the power of radiomics analysis in differentiating between patients with acute myocardial infarction (MI) and patients with stable or no CAD. In their prospective case-control study radiomics analysis of PCAT around the proximal right coronary artery was performed on patients with acute MI (*n* = 60), with stable CAD (*n* = 60), and a control group without CAD (*n* = 60). 1,103 radiomics features were extracted, 20.3% of which differed significantly between MI patients and the control group while 16.5% differed between MI and stable CAD patients. Following up on these findings the study showed that a machine learning model integrating radiomics parameters into clinical features and PCAT attenuation proved to be better at detecting acute MI patients compared to models only using clinical features with or without PCAT attenuation (28). This strengthens our suggestion, that deeper texture analysis will provide additional information as outlined in our study and might offer potential additional imaging biomarkers in the future.

In line with these findings, Oikonomou et al. analyzed radiomics features of PCAT in 101 patients with MACE within five years after CCTA and compared the results to 101 patients from their control group. Again, radiomics analysis, namely the fat radiomics profile which was significantly higher in patients with acute MI, improved the prediction of MACE significantly compared to conventional risk factors including high-risk plaque features. They also showed that tissue inflammation was best correlated to adipose tissue wavelet-transformed mean attenuation, while fibrosis and vascularity were better matched to higher-order texture features (14). While our study did not focus on MACE prediction in general but rather investigated the association of the specific risk factor hypercholesterolemia to structural changes of PCAT, the results of the studies described above show that radiomics analysis of PCAT can offer great accuracy in risk prediction and can distinguish between different changes such as inflammation, fibrosis, and vascularity, suggesting there can be a benefit in this more specific analysis of PCAT.

The differences in the power of prediction of a future acute coronary syndrome (ACS) between radiomics analysis of PCAT and conventional plaque characteristics were investigated by Shang et al. In this study, 90 patients with ACS within three years after receiving CCTA were identified, PCAT surrounding the responsible lesion was segmented and analyzed, and 14 conventional plaque characteristics were collected. A control group of 90 patients without ACS was analyzed in the same way. The derived radiomics score consisting of 14 features outperformed the conventional plaque score significantly in the identification of patients who suffered from ACS within three years (AUC = 0.826, 0.811 radiomics score; AUC = 0.699, 0.640 plaque score, in training and test set, respectively) (29). This shows once more the great benefit the identification of imaging biomarkers can offer for improving cardiac risk prediction.

However, there are still limitations to this study. First, the execution as a single-center study with a small study population must be brought to attention. The reason for this study design was mainly the very novel implementation of the PCCT scanner. Hence, this study misses addressing a main disadvantage of radiomics analysis namely the insufficient reproducibility. Also due to the novelty of the PCCT scanner and the therefore regularly necessary software updates, the voltage of the scans of the test and the validation collective differed slightly. Nevertheless, other studies in our institution have focused on the impact of the detector type (EICT vs. PCCT) on radiomics analysis of the left ventricular myocardium, outlining a possibly deeper insight into the texture by PCCT (30). The capability of PCCT of constructing monochromatic reconstructions with different keV could further improve feature stability, especially regarding second order texture features and future studies should focus on exploring the clinical applicability of this aspect (31). The high stability of radiomics features when using PCCT could recently be demonstrated with a phantom model, pointing towards a probable improvement in comparability through the implementation of PCCT (8). The aim of our study, however, was to outline a possible influence of a specific cardiovascular risk factor on pericoronary adipose tissue and did not focus on a feature stability analysis. Furthermore, clinical data were collected through questionnaires and if available through medical records, hence some clinical data might not have been collected fully. To address this limitation, patients with insufficient questionnaires and no medical record were not included in this study. Additionally, the influence of other risk factors on structural changes in PCAT texture, such as hypertension or nicotine abuse, was not considered thoroughly in this study. There could be a possible contributing effect of the other cardiovascular risk factors on PCAT texture although a statistically significant difference of other cardiovascular risk factors between both groups could be excluded. To address these limitations, further studies should focus on pericoronary adipose tissue in a multicenter prospective approach with a larger study population in the future.

This study is to our knowledge the first study outlining a dedicated influence of hypercholesterolemia on the pericoronary adipose tissue in a patient population on the newly established PCCT. These preliminary findings indicate a higher mean density of PCAT in affected patients in correlation with specific texture features representing a predominantly greater concentration of high-density values, pointing towards a wider potential of radiomics analysis as a tool for finding and developing non-invasive imaging biomarkers for arteriosclerosis and other cardiovascular diseases.

## Data Availability

The raw data supporting the conclusions of this article will be made available by the authors, without undue reservation.
